# A new nomogram and risk classification system for predicting survival in small cell lung cancer patients diagnosed with brain metastasis: a large population-based study

**DOI:** 10.1186/s12885-021-08384-5

**Published:** 2021-05-29

**Authors:** Qinge Shan, Jianxiang Shi, Xiaohui Wang, Jun Guo, Xiao Han, Zhehai Wang, Haiyong Wang

**Affiliations:** 1 Department of Internal Medicine-Oncology, Shandong Cancer Hospital and Institute, Shandong First Medical University and Shandong Academy of Medical Sciences, Jinan, 250117 Shandong China; 2 Henan Academy of Medical and Pharmaceutical Sciences, Precision Medicine Center, Zhengzhou University, Zhenzhou, Henan China; 3 Research Service Office, Shandong Liaocheng People’s Hospital, Liaocheng, Shandong China

**Keywords:** Small cell lung cancer, Brain metastasis, Survival, Prediction, Nomogram

## Abstract

**Background:**

The prognosis of patients with small cell lung cancer (SCLC) is poor, most of them are in the extensive stage at the time of diagnosis, and are prone to brain metastasis. In this study, we established a nomogram combined with some clinical parameters to predict the survival of SCLC patients with brain metastasis.

**Methods:**

The 3522 eligible patients selected from the SEER database between 2010 and 2015 were randomly divided into training cohort and validation cohort. Univariate and multivariate Cox regression analysis were used to evaluate the ability of each parameter to predict OS. The regression coefficients obtained in multivariate analysis were visualized in the form of nomogram, thus a new nomogram and risk classification system were established. The calibration curves were used to verify the model. And ROC curves were used to evaluate the discrimination ability of the newly constructed nomogram. Survival curves were made by Kaplan-Meier method and compared by Log rank test.

**Results:**

Univariate and multivariate analysis showed that age, race, sex, T stage, N stage and marital status were independent prognostic factors and were included in the predictive model. The calibration curves showed that the predicted value of the 1- and 3-year survival rate by the nomogram was in good agreement with the actual observed value of the 1- and 3-year survival rate. And, the ROC curves implied the good discrimination ability of the predictive model. In addition, the results showed that in the total cohort, training cohort, and validation cohort, the prognosis of the low-risk group was better than that of the high-risk group.

**Conclusions:**

We established a nomogram and a corresponding risk classification system to predict OS in SCLC patients with brain metastasis. This model could help clinicians make clinical decisions and stratify treatment for patients.

**Supplementary Information:**

The online version contains supplementary material available at 10.1186/s12885-021-08384-5.

## Background

Small cell lung cancer (SCLC) accounts for 13% of all lung cancer, and the prognosis is very poor, most of which are in the stage of extensive disease [1, 2]. Moreover, patients with SCLC has a high propensity to develop brain metastasis at initial diagnosis [3]. For patients with brain metastasis, whole brain radiotherapy has become the first choice because of the radio-sensitivity of SCLC and the risks associated with local treatment [4, 5]. Although patients received prophylactic cranial irradiation, most patients relapsed with brain metastasis [6, 7]. CASPIAN study [8] found that the median overall survival of extensive stage small cell lung cancer (ES-SCLC) patients treated with durvalumab combined with chemotherapy as first-line treatment was 13.0 months, and about 34% of the patients survived at 18 months, which was better than that of chemotherapy alone. The updated results of the CASPIAN study showed that compared with the chemotherapy group, the durvalumab plus chemotherapy group continued to show OS benefits, with 2-year survival rates of 22 and 14.4% in the combination group and chemotherapy group, respectively [9]. The efficacy of immune checkpoint inhibitors in the treatment of brain metastasis from melanoma and non-small cell lung cancer has been confirmed, but there is insufficient evidence of the feasibility of SCLC brain metastasis immunotherapy [10–12].

Nomograms have been widely applied to predict the survival rate of cancer patients, and compared with the traditional TNM staging system, it is more accurate for personalized prognosis prediction [13–17]. Therefore, in this study, we analyzed the data extracted from the Surveillance, Epidemiology, and End Results (SEER) database of SCLC patients diagnosed with brain metastasis between 2010 and 2015. Our aim was to identify the key factors affecting the prognosis of SCLC patients diagnosed with brain metastasis and to establish and validate a nomogram that could predict the prognosis of these patients.

## Methods

### Patient selection

We selected patients diagnosed with small cell lung cancer between 2010 and 2015 from the SEER database, which was based on the Department of Cancer Control and Population Sciences of the National Cancer Institute (NCI) [18]. The inclusion criteria for this study were as follows: patients were pathologically diagnosed as SCLC; only one primary tumor; patients with brain metastasis confirmed at initial diagnosis. Patients with incomplete clinical information were excluded, such as age, race, sex, T stage, N stage, marriage, and survival time. In the end, we selected 3522 eligible cases for retrospective analysis.

### Ethics statement

Because the data extracted from the SEER database in this study did not contain personally identifiable information, informed consent and ethical proof were not required.

### Statistical analysis

The main endpoint of this study was overall survival (OS). In this study, 3522 patients were randomly divided into training cohort and validation cohort, with a ratio of 7:3. The data of the training cohort was used not only to establish the prediction model, but also to construct the nomogram and risk classification system. The data in the validation cohort was utilized to validate the model built by the training cohort.

Univariate and multivariate cox regression analysis were used to determine independent prognostic variables affecting OS. Based on the factors contained in the final model, the nomogram and risk classification system were established. The calibration curves were used to evaluate the calibration of the prediction model by comparing the predicted survival time and observed survival rate of 1- and 3-year. And ROC curves were used to evaluate the discrimination ability of the newly constructed nomogram. Furthermore, the establishment of the risk classification system was based on the total score of each patient in the validation cohort, and all patients were divided into low-risk and high-risk prognosis groups. Survival curves were made by Kaplan-Meier method and compared by Log rank test*.* Data analysis used R software version 3.4.3 (R Foundation) and Statistical Product and Service Solutions (SPSS) version 22.0. All tests were two-tailed and *P* < 0.05 was considered statistically significant, except for univariate regression analysis.

## Results

### Patients characteristics

In this study, 3522 patients included were randomly divided into training cohort (*n* = 2466, 70%) and validation cohort (*n* = 1056, 30%). Of the total cohort, training cohort, and validation cohort, the number of patients aged 50–69 (*n* = 2228, *n* = 1567, *n* = 661, respectively) was the largest, nearly two-thirds of the number of patients in each group. Male patients (*n* = 1877) were slightly more than female patients (*n* = 1645), and the ratio of male to female in all cohorts was 1.14:1. Regard to T staging, T4 staging was the most in total cohort, training cohort and validation cohort (*n* = 1450, *n* = 1034, *n* = 416, respectively), followed by T2 staging (*n* = 878, *n* = 592, *n* = 286, respectively), and T0 staging (*n* = 49, *n* = 34, *n* = 15, respectively) was the least. In the N stage of the total, training and the validation cohorts, the highest proportion was N2 stage (*n* = 1928, *n* = 1362, *n* = 566, respectively), up to more than half, followed by N3 stage. In each cohort, the number of unmarried and married people (1713 vs. 1809 in total cohort; 1191 vs. 1275 in training cohort; 522 vs. 534 in validation cohort) was similar. Baseline characteristics were balanced between training and validation cohort, as detailed in Table 1.

**Table 1:** Baseline clinicopathological characteristics of all patients and those in the training and validation cohort

Variables	All cohorts (*n* = 3522)	Training cohort (*n* = 2466)	Validation cohort (*n* = 1056)
**Age**			
30–49	153 (4.3%)	100 (4.1%)	53 (5.0%)
50–69	2228 (63.3%)	1567 (63.5%)	661 (62.6%)
≥ 70	1141 (32.4%)	799 (32.4%)	342 (32.4%)
**Race**			
White	2972 (84.4%)	2079 (84.3%)	893 (84.6%)
Black	391 (11.1%)	275 (11.2%)	116 (11.0%)
Others	159 (4.5%)	112 (4.5%)	47 (4.4%)
**Sex**			
Male	1877 (53.3%)	1292 (52.4%)	585 (55.4%)
Female	1645 (46.7%)	1174 (47.6%)	471 (44.6%)
**T stage**			
T0	49 (1.4%)	34 (1.4%)	15 (1.4%)
T1	366 (10.4%)	246 (10.0%)	120 (11.4%)
T2	878 (24.9%)	592 (24.0%)	286 (27.1%)
T3	779 (22.1%)	560 (22.7%)	219 (20.7%)
T4	1450 (41.2%)	1034 (41.9%)	416 (39.4%)
**N stage**			
N0	487 (13.8%)	336 (13.6%)	151 (14.3%)
N1	269 (7.6%)	186 (7.6%)	83 (7.9%)
N2	1928 (54.8%)	1362 (55.2%)	566 (53.6%)
N3	838 (23.8%)	582 (23.6%)	256 (24.2%)
**Marriage**			
Unmarried	1713 (48.6%)	1191 (48.3%)	522 (49.4%)
Married	1809 (51.4%)	1275 (51.7%)	534 (50.6%)

### Univariate and multivariate analysis to determine the factors that predict OS

Univariate and multivariate cox regression analysis were used to screen the independent prognostic factors of OS in patients with brain metastasis of small cell lung cancer. Univariate regression analysis showed the effects of age (*P* < 0.001), race (*P* = 0.060), sex (*P* = 0.030), T stage (*P* < 0.001), N stage (*P* < 0.001) and marital status (*P* < 0.001) on the prognosis of patients with brain metastasis of SCLC. Multivariate cox regression analysis further analyzed the factors of a *P* < 0.1 in univariate cox regression analysis. Multivariate analysis demonstrated that age, race, sex, T stage, N stage and marital status were independent prognostic factors and were included in the predictive model. Table 2 showed the results of univariate and multivariate cox analysis to evaluate the prognostic factors of OS. And the flow chart of this study was shown in Figure S1.

**Table 2:** Univariate and multivariate cox analyses to evaluate the prognostic factors for OS

Variable	Univariate analyses			Multivariate analyses		
	HR	95% CI	*P*	HR	95% CI	*P*
**Age**			< 0.001			
30–49	Reference			Reference		
50–69	1.146	0.928–1.414	0.205	1.198	0.969–1.480	0.095
≥ 70	1.626	1.310–2.019	< 0.001	1.767	1.421–2.198	< 0.001
**Race**			0.060			
White	Reference			Reference		
Black	1.000	0.873–1.144	0.997	0.962	0.839–1.103	0.579
Others	0.776	0.630–0.955	0.016	0.734	0.595–0.905	0.004
**Sex**			0.030			
Male	Reference			Reference		
Female	0.911	0.837–0.992	0.0311	0.887	0.8135–0.966	0.006
**T stage**			< 0.001			
T0	Reference			Reference		
T1	1.449	0.970–2.164	0.070	1.343	0.898–2.008	0.151
T2	1.582	1.074–2.330	0.020	1.451	0.984–2.140	0.060
T3	1.830	1.242–2.698	0.002	1.664	1.127–2.456	0.010
T4	1.775	1.210–2.603	0.003	1.623	1.104–2.387	0.014
**N stage**			< 0.001			
N0	Reference			Reference		
N1	0.964	0.795–1.168	0.707	0.931	0.768–1.129	0.469
N2	1.242	1.090–1.414	0.001	1.230	1.079–1.402	0.002
N3	1.236	1.068–1.431	0.005	1.220	1.052–1.415	0.009
**Marriage**			< 0.001			
Unmarried	Reference			Reference		
Married	0.862	0.792–0.938	< 0.001	0.851	0.780–0.928	< 0.001

### Establishment and verification of predictive nomogram

**Fig. 1:**
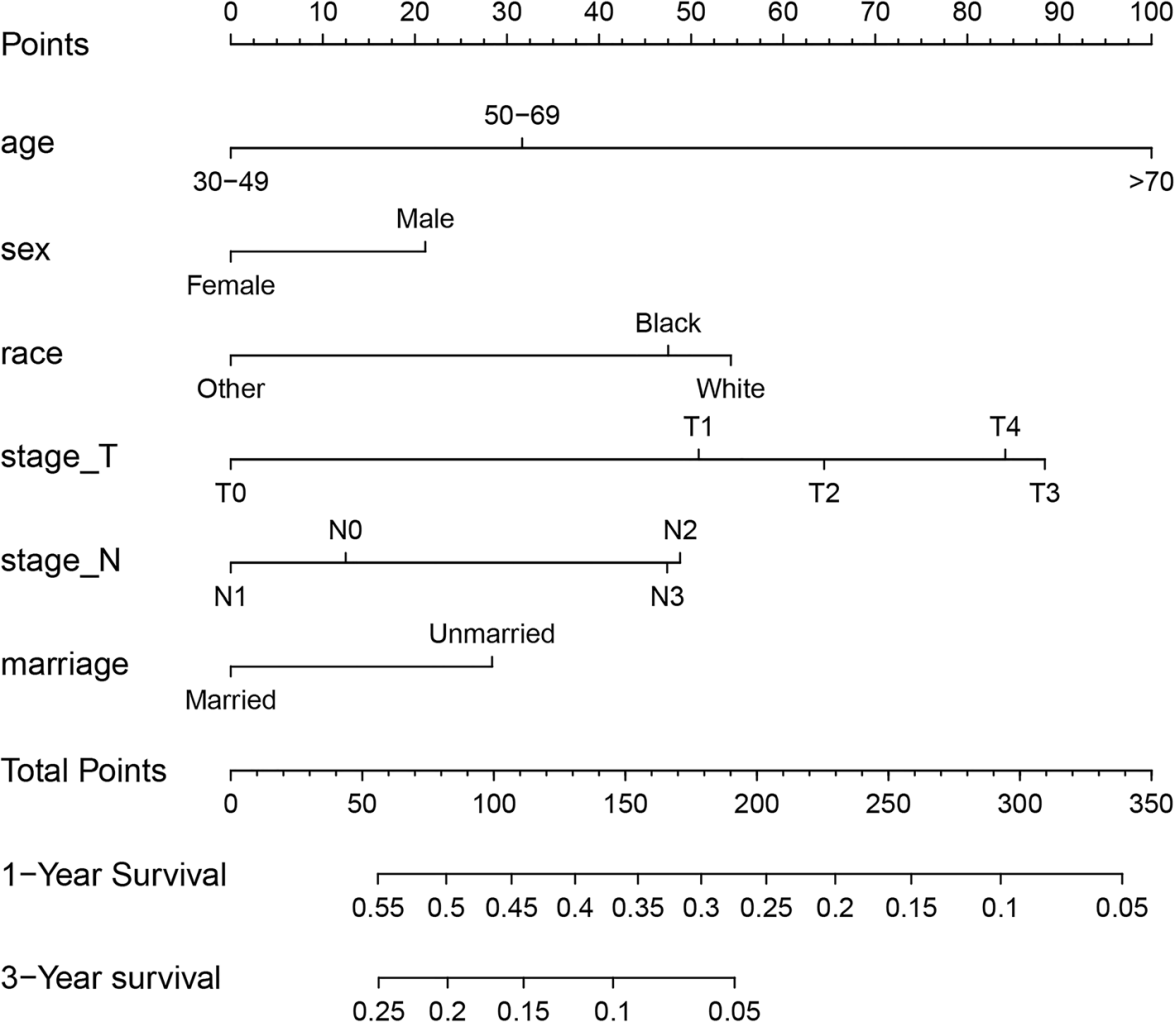
A nomogram for prediction of 1-, and 3-year OS rates of SCLC patients diagnosed with brain metastasis

The prediction model was actually visualized in the form of a nomogram, and a new nomogram was established. As shown in Fig. 1, each factor had a score on the point scale. A straight line could be drawn to determine the estimated prognosis probability at each time point by adding the total score and locating it on the total point scale.

**Fig. 2:**
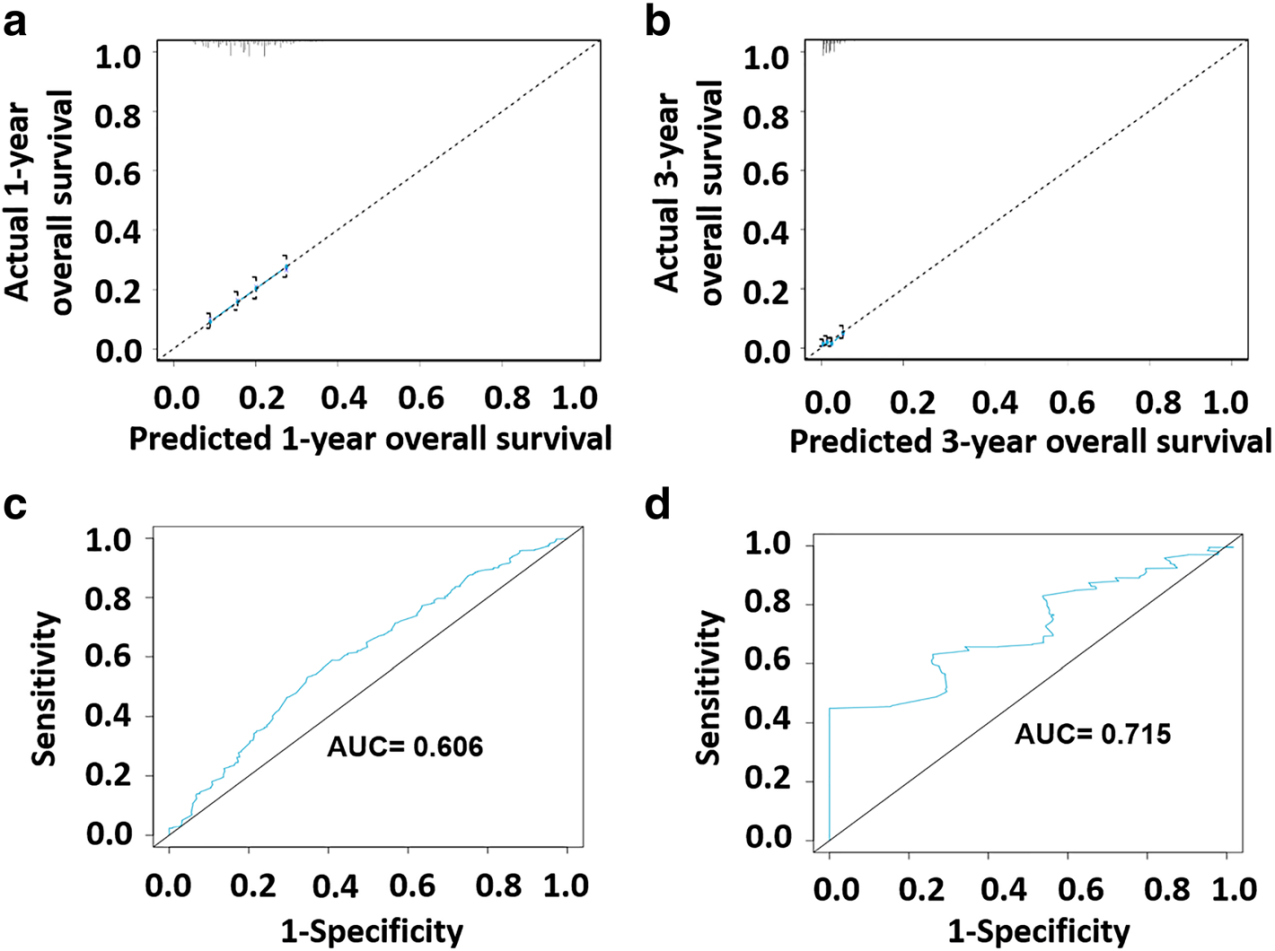
Calibration curves and receiver operating characteristic (ROC) Curves. **a** Calibration curves showing the probability of 1-year OS between the nomogram prediction and the actual observation; **b** Calibration curves showing the probability of 3-year OS between the nomogram prediction and the actual observation. The prediction probability of the nomogram for OS was plotted on the X-axis, and the actual probability was plotted on the Y-axis. **c** The ROC curve of nomogram for predicting 1-year survival rate and area under curve (AUC) = 0.606; **d** The ROC curve of nomogram for predicting 3-year survival rate and AUC = 0.715

In the calibration curves, the OS occurrence probability predicted by nomogram was compared with the observed 1- and 3-year OS occurrence probability. In a well-calibrated model, the prediction will fall on the diagonal of 45-degree. As can be seen from Fig. 2, the prediction of the 1-and 3-year survival rate by the nomogram was in good agreement with the actual observation of the 1-and 3-year survival rate. In addition, we used receiver operating characteristic (ROC) curves to verify the discrimination of the prediction model. As shown in Fig. 2, the area under curve (AUC) of 1-year and 3-year survival rates was 0.606 and 0.715, respectively.

### Risk classification system

**Fig. 3:**
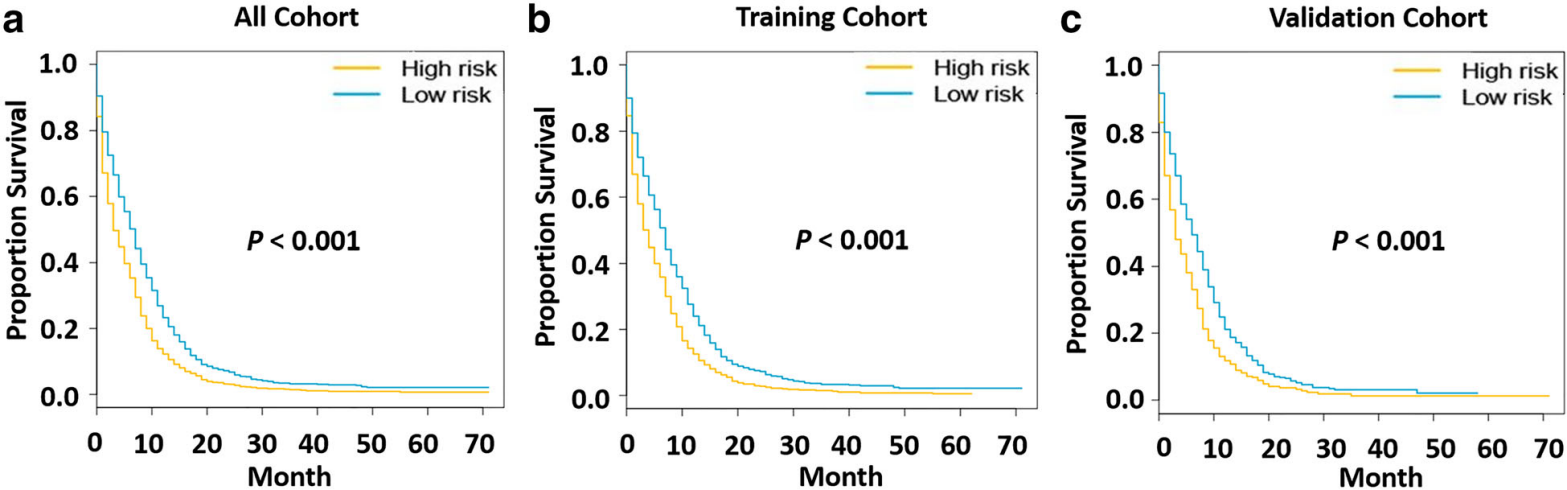
Survival curves of high-and low-risk groups in each cohort. **a** Survival curves of high-risk group and low-risk group in the total cohort. **b** Survival curves of high-risk group and low-risk group in the training cohort. **c** Survival curves of high-risk group and low-risk group in the validation cohort

A risk classification system was developed based on the total score of each patient in the training cohort generated by nomogram. According to the established risk classification system, all patients were divided into two prognostic groups of low risk (risk score: 0–0.99) and high risk (risk score: 1.00–1.74), and the number of cases in the two groups was similar. Then, we plotted Kaplan-Meier curves of OS for each cohort of low-risk and high-risk groups (Fig. 3). In the training cohort, we found that the prognosis of the low-risk group was better than that of the high-risk group (*P <* 0.001). At the same time, in the validation cohort, it could be found that the low-risk group had a better OS (*P <* 0.001). In addition, the results also showed that the low-risk group had a better prognosis in the total cohort (*P <* 0.001).

## Discussion

In this study, a nomogram and risk classification system were established and verified to predict the prognosis of patients with brain metastasis of SCLC. The predictors of this nomogram included sex, age, race, T stage, N stage and marital status. We verified the model with different statistical methods to prove that the model had a good prediction ability. Finally, we found that the nomogram predictions of 1-year and 3-year OS curves were in good agreement with the actual observations.

Some studies showed that age, gender, race and TNM stage were significantly correlated with OS in patients with SCLC [19, 20]. A study conducted by Ou et al. [21] showed that being unmarried (HR = 1.179, *P <* 0.001) was an independent prognostic factor for ES-SCLC. Our results were consistent with these studies. In this model, age, sex, race, T stage, N stage and marital status were retained after backward selection.

Advances in genome sequencing of SCLC suggested that SCLC was a relatively heterogeneous disease characterized by mutations in *TP53*, *RB1*, and *Notch* genes, as well as copy number variations in chromosome 3p, *JAK2*, *FGFR1*, and *MYC* [22–24]. Almost 100% of SCLC patients have mutations or functional changes in *P53* and *RB1*, and a considerable proportion of people have changes in *MYC* function [22, 25]. Therefore, the prognosis of patients at the same stage in the traditional staging system varied greatly. Obviously, the heterogeneity of SCLC determined that the traditional staging method to predict the prognosis of SCLC was not appropriate to some extent. At present, studies have shown that nomogram could be used to predict the prognosis of SCLC patients. Pan et al. [26] established and validated a nomogram with seven predictors to predict the prognosis of SCLC patients. Moreover, the study by Pan et al. [26] revealed that the model could predict the survival probability of patients with SCLC more accurately than the existing staging system. Xie et al. [27] demonstrated that the effect of nomogram combined with hematological indicators in predicting the prognosis of SCLC was better than that of the existing prediction models. A new nomogram prognostic model based on a large sample of SCLC patients also showed that the nomogram had better predictive power than previous models [28].

Nowadays, nomograms have been established to predict the prognosis of non-small cell lung cancer (NSCLC) patients with brain metastasis. Won et al. [29] established a nomogram including histological type, N stage, T stage and smoking status to predict brain metastasis in NSCLC patients. Another nomogram for predicting brain metastasis in NSCLC patients included predictors of histological type, tumor size, and number of metastatic lymph nodes [30]. In addition, some researchers had used nomogram to predict the survival of some special types of non-small cell lung cancer, such as pulmonary invasive mucinous adenocarcinoma. A study [31] had shown that the prognosis of pulmonary invasive mucinous adenocarcinoma was related to age, differentiation, TNM stage and treatment, and a new nomogram which could predict the prognosis had been established. Nevertheless, up to now, the nomogram has not been applied to predict brain metastasis of SCLC. Therefore, we extracted data from SEER database to establish and validate a novel predictive model for predicting the prognosis of SCLC patients with brain metastasis. The nomogram predictive model for predicting brain metastasis of SCLC might not only help to clarify treatment stratification and efficacy evaluation, but also contribute to establish the inclusion criteria of clinical trials in SCLC patients with brain metastasis. Using this prediction model, researchers and clinicians could easily predict the survival probability of each SCLC patient with brain metastasis.

In addition, we used validation cohort to verify the discrimination ability and stability of this model. Nomogram verification was very important not only to determine the universality of the nomogram, but also to prevent the model from overestimating the predictability of the sample [32]. The results showed that this nomogram had the best consistency between prognosis prediction and actual observation. Therefore, the nomogram established in this study provided a good prediction model for predicting OS in SCLC patients with brain metastasis.

Limitations must be acknowledged in this study. First, this study was a retrospective study with its own limitations, such as unavoidable selection bias. Secondly, due to the limitation of SEER database, the data of smoking, socioeconomic status, general health status, grade, gene mutation and treatment regimen cannot be obtained, which hindered the further analysis of prognosis. Third, many known prognostic factors of SCLC were not included in this study, such as hematological markers and hematological markers [27]. Although the nomogram and risk classification were built using a large cohort and verified in the validation cohort, more external validation of the prediction model is still necessary for future applications. Despite these limitations, this study was the first to develop a nomogram prediction model for predicting survival in SCLC patients with brain metastasis.

## Conclusion

We established a nomogram and a corresponding risk classification system to predict OS in SCLC patients with brain metastasis. Through the verification of the model, it was proved that the model had good performance. This model could help clinicians make clinical decisions and stratify treatment for patients. At the same time, it could provide a basis for researchers to determine reasonable stratification parameters in future clinical trials. Of course, further studies are needed to confirm its application in SCLC patients with brain metastasis.

## Supplementary Information

**Additional file 1: Figure S1.** Flow chart of model development.

## Data Availability

The datasets analyzed during the current study are available from the corresponding author on reasonable request.

## Abbreviations

SCLC: small cell lung cancer
SEER: Surveillance, Epidemiology, and End Results
OS: Overall survival
ES-SCLC: Extensive stage small cell lung cancer
NSCLC: Non-small cell lung cancer
AUC: Area under curve
ROC curve: Receiver operating characteristic curve
