# Supplementary figures and images for: A new nomogram and risk classification system for predicting survival in small cell lung cancer patients diagnosed with brain metastasis: a large population-based study
Source: BMC Cancer. 2021 May 29;21:640. doi: 10.1186/s12885-021-08384-5 (PMC8164795; doi:10.1186/s12885-021-08384-5)

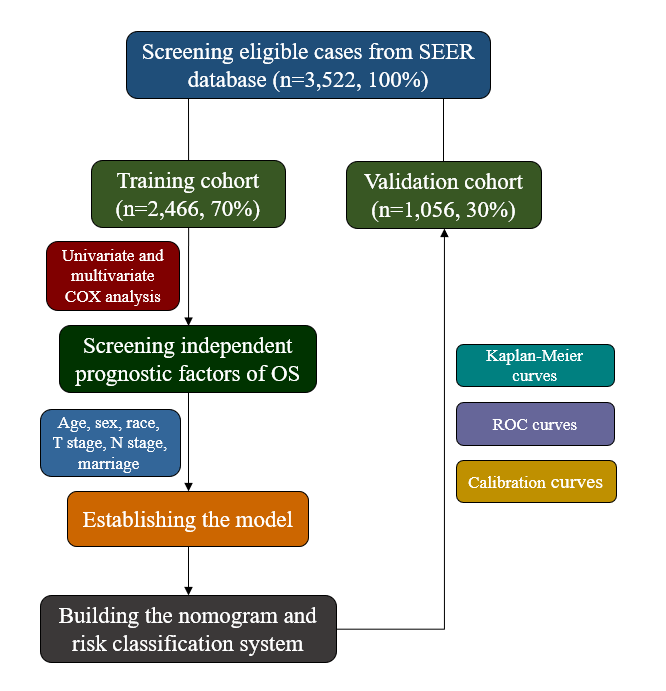

Supplement: Supplementary file 1 — Additional file 1: Figure S1. Flow chart of model development. [file 12885_2021_8384_MOESM1_ESM.tif]
